# Top-down Mass Spectrometry Analysis of Human Serum Autoantibody Antigen-Binding Fragments

**DOI:** 10.1038/s41598-018-38380-y

**Published:** 2019-02-20

**Authors:** Zhe Wang, Xiaowen Liu, Jennifer Muther, Judith A. James, Kenneth Smith, Si Wu

**Affiliations:** 1 Department of Chemistry and Biochemistry, University of Oklahoma, Norman, OK 73019 USA; 2 Department of BioHealth Informatics, Indiana University-Purdue University Indianapolis, Indianapolis, IN 46202 USA; 3 Department of Arthritis and Clinical Immunology, Oklahoma Medical Research Foundation, Oklahoma City, OK 73104 USA; 4 Departments of Medicine and Pathology, University of Oklahoma Health Sciences Center, Oklahoma City, OK 73104 USA

## Abstract

Detecting autoimmune diseases at an early stage is crucial for effective treatment and disease management to slow disease progression and prevent irreversible organ damage. In many autoimmune diseases, disease-specific autoantibodies are produced by B cells in response to soluble autoantigens due to defects in B cell tolerance mechanisms. Autoantibodies accrue early in disease development, and several are so disease-specific they serve as classification criteria. In this study, we established a high-throughput, sensitive, intact serum autoantibody analysis platform based on the optimization of a one dimensional ultra-high-pressure liquid chromatography top-down mass spectrometry platform (1D UPLC-TDMS). This approach has been successfully applied to a 12 standard monoclonal antibody antigen-binding fragment (Fab) mixture, demonstrating the feasibility to separate and sequence intact antibodies with high sequence coverage and high sensitivity. We then applied the optimized platform to characterize total serum antibody Fabs in a systemic lupus erythematosus (SLE) patient sample and compared it to healthy control samples. From this analysis, we show that the SLE sample has many dominant antibody Fab-related mass features unlike the healthy controls. To our knowledge, this is the first top-down demonstration of serum autoantibody pool analysis. Our proposed approach holds great promise for discovering novel serum autoantibody biomarkers that are of interest for diagnosis, prognosis, and tolerance induction, as well as improving our understanding of pathogenic autoimmune processes.

## Introduction

Autoimmune diseases are a leading cause of death and disability in young minority women and collectively affecting more than 23.5 million Americans^1^. More than 80 different autoimmune diseases exist and many share similar symptoms, making clinical diagnosis of autoimmune diseases difficult^2^. Most autoimmune diseases are chronic conditions which can be controlled to varying extents by medication, but there is no permanent cure and these medications often have significant toxicities^3,4^. Therefore, detecting systematic autoimmune diseases at an early stage is crucial for effective treatment and disease management to slow disease progression and prevent irreversible organ damage. However, this remains a significant clinical challenge due to the lack of unique biomarkers with both specificity and sensitivity^2^.

Autoantibodies are a hallmark of many autoimmune diseases and can be present in serum years before clinical symptoms arise^5^ and are occasionally present even in healthy individuals^6^. Current analysis approaches (*e*.*g*., the enzyme-linked immunosorbent assay, ELISA) only measure total concentrations of autoantigen specific autoantibodies that are often polyclonal and may contain highly homologous clonal sequences^7,8^. On the other hand, the presence of specific monoclonal autoantibodies in patients with autoimmune diseases is of interest for diagnosis, prognosis, drug targets, and for our understanding of various disease processes. DNA deep sequencing of the B cell antibody repertoire can be used to analyze humoral immune responses^9^, but few of the detected sequences are represented in the circulating pool of serum immunoglobulins, and it is essentially impossible to determine which sequences are specific to an antigen of interest. Therefore, to elucidate functionally relevant autoantibodies that mediate autoimmune responses, protein-level characterization of autoantibodies in the patient serum (*i*.*e*., proteomics) is needed to precisely determine which of these autoantibody clones are predictive of autoimmune disease progression.

Mass spectrometry-based proteomics techniques have been used for the detection and characterization of serum monoclonal antibodies. Several bottom-up and middle-down approaches have been developed to identify autoantibodies in serum^10–14^. These approaches often start with affinity purification of polyclonal autoantibodies from human serum with an autoantigen of interest. The purified antibodies are then digested with proteases such as trypsin to produce peptide fragments that are analyzed by LC-MS/MS. Identification of the peptide sequences corresponding to antibody fragments can be performed either with reference databases or through *de novo* sequencing. However, there are inherent challenges with bottom-up approaches for serum antibody analysis. Serum autoantibodies are likely to be highly homologous with very similar sequences from common V gene families. Bottom-up proteomics on serum autoantibodies, starting with digested peptides, will result in a pool of peptides with both shared and non-shared sequences. Even assuming 100% sequence coverage (which is nearly impossible to generate with bottom-up approaches), without additional information, bottom-up MS is unable to identify the precise coordination of individual sequences for each IgG.

Top-down proteomics has unique advantages in analyzing proteoforms with sequence variations and post-translational modifications (PTMs) because it analyzes intact proteoforms rather than short peptides^15–18^. Recent developments in MS instrumentation and protein separation have paved the way for proteome-wide analysis of complex, including intact monoclonal antibodies^13,19–23^. A top-down proteomics approach (*i*.*e*., miRAMM) has been demonstrated for monitoring the light chain of a single monoclonal therapeutic IgG in spiked-in serum. Recently, the miRAMM was applied with the ultrahigh resolution MS (*i*.*e*., 21T FTICR-MS) to analyze several spiked-in monoclonal antibodies in human serum offering the high mass accuracy and high sequence coverage^21^. However, because multiple autoantigens co-exist in autoimmune diseases, sera of autoimmune disease patients are very complex, likely containing at least hundreds of highly homologous monoclonal autoantibodies. Thus, miRAMM or similar approaches cannot be directly applied to analyze serum autoantibodies without significantly advancing the analytical capability to separate many highly homologous autoantibodies from the serum antibody background.

With top-down proteomics, reversed phase liquid chromatography (RPLC) is the most commonly applied high-throughput separation approach that can be coupled directly online with MS^15^. Similar to bottom-up MS, longer column and higher pressure pumps are used to improve the peak capacity of RPLC separation^15,24,25^. We have previously reported a single-dimension top-down proteomics platform analysis with home-built nanoflow columns and Waters nanoAquity pumps (maximum pressure 10,000 psi, operation pressure 7,000 psi)^25–27^. We have applied the platform to the full characterization of the variable regions of two pharmaceutical monoclonal antibodies with sensitivity comparable to the bottom-up standard^28^. In this study, we optimized an automatic single-dimension RPLC platform through a custom-modified ultra-high pressure LC system (UPLC, maximum pressure 14,000 psi, operation pressure 10,000 psi) to improve the separation of highly homologous autoantibodies (*i*.*e*., intact Fab, light chain and heavy chain of the Fab portion) in serum samples. This approach has been successfully applied to a 12 standard monoclonal antibody Fab mixture, demonstrating the feasibility to separate and sequence intact antibodies with high sequence coverage and high sensitivity. We then applied the optimized platform to characterize serum autoantibody Fabs in a systemic lupus erythematosus (SLE) patient sample compared to healthy control samples, showing that 86 dominant antibody Fab related mass features are observed in the SLE sample, which is the first top-down demonstration of a serum autoantibody pool analysis.

## Results

### Optimization of the UPLC-TD-HRMS platform

The 1D UPLC-TD-HRMS was developed and optimized using a Waters NanoAcquity HPLC system with the maximum pressure of 10,000 psi in our previous work^25–27,29–31^. However, the routine operational pressure of the system was limited to 7,000 psi due to commercial system limitations and automation. The operational pressure often changes with the organic components in the elution buffer, and the run would be interrupted when the pressure reached the maximum pressure. Recently, Shen *et al*. demonstrated high peak capacities in long-column RPLC separation at ultra-high pressure operation pressure (maximum operation pressure at 14,000 psi) using a manually operated constant pressure syringe pumps with customized gradient mixers^24^. To improve the throughput for long-column RPLC separation at higher operation pressure limits, we modified a commercially available normal-flow system (*e*.*g*., Thermo Accela pumps, maximum pressure 14,000 psi) through the customized splitting system to establish an automatic UPLC system that can be routinely operated at a pressure higher than 10,000 psi for long-column nano-LC separations (operation flow rate between 100 nL/min to 400 nL/min). (Fig. S1).

**Figure 1:**
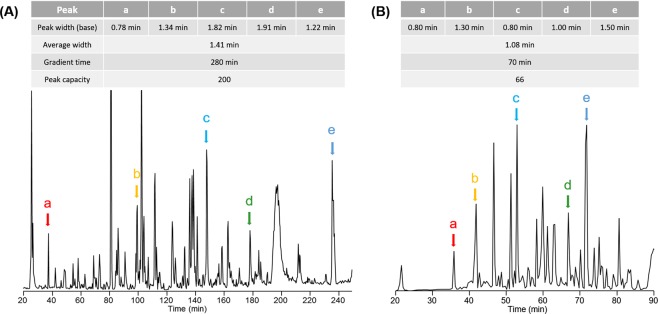
Two LC-MS runs of intact *E*. *coli* lysate proteins with a 280 minute gradient (**A**) and a 70 minute gradient (**B**). Five randomly chosen peaks (a–e) are used for the calculation of the peak capacity.

The elution gradient was optimized using a custom packed C5 long column (C5, 100 cm length, 360 µm o.d., 75 µm i.d.). To evaluate the system performance, ten micrograms of *E*. *coli* lysate proteins were loaded on the column and an elution gradient from 10% to 70% of mobile phase B was applied over 70 minutes and 280 minutes separately. The peak capacities with different gradient times were calculated by comparing the base peak widths of five randomly selected proteins from the LC/MS runs^32^ (Fig. 1). The average base peak widths were 1.08 minutes for a 70-minute gradient (Fig. 1B) and 1.41 minutes for a 280-minute gradient (Fig. 1A), respectively. The peak capacity of a 70-minute run was calculated as 66, and the peak capacity of a 280-minute run was 200. Our results suggested that elution peak widths did not increase remarkably with longer gradient time. Similar results were observed with a 200-minute elution gradient. In addition, we did a 350-minute gradient for complex separation and we noticed that peaks are significantly broadening. One possible reason is that the operation of longer columns at ultra-high pressures can partially overcome the resolution loss from the diffusion with longer separation time^24^. However, longer gradient time than 280 minutes will not significantly improve the separation resolution. Based on the results, we here chose 280 minutes as the gradient time (10–70% of mobile phase B) for the separation of 12-Fab mixture and Fab fragments enriched from human serum samples.

### UPLC-TD-HRMS analysis of a 12-antibody mixture

**Figure 2:**
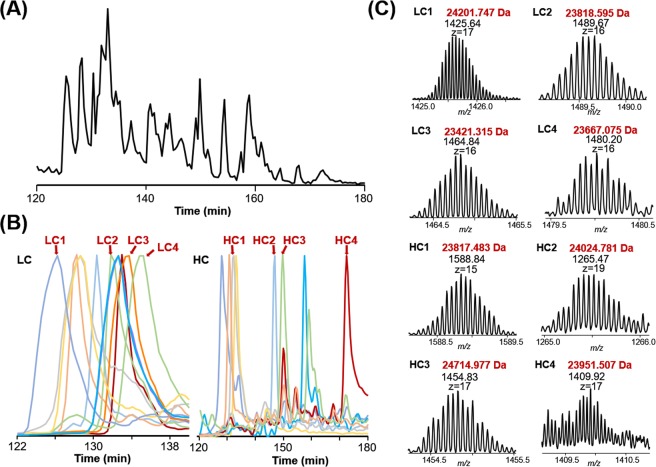
The UPLC-TD-HRMS analysis of the reduced 12 intact Fab mixture. (**A**) The base peak chromatogram (BPC) over the *m/z* range of 550 to 2,000; (**B**) selected extracted ion chromatograms (EICs) of light chains (left) and heavy chains (right); (**C**) MS spectra of representative light chains and 4 heavy chains.

To evaluate the separation performance of intact antibody Fabs using the optimized UPLC-TD-HRMS platform, we papain-digested 12 fully human monoclonal antibodies and enriched the Fab fragments using protein A agarose beads. These 12 Fabs were mixed in equal quantities, and eight micrograms of the 12-Fab mixture was reduced by TCEP before being loaded onto the C5 column for top-down MS analysis. Base peak chromatograms (BPC) of the separation of the 12-Fab mixture (Fig. 2A) as well as the extracted ion chromatograms (EICs) (Fig. 2B) of the light chain and heavy chain for each Fab fragment are shown. Overall, Fab light chains eluted earlier (between 27% and 30% B) compared with Fab heavy chains (between 27% and 38% B). Combined with high-resolution MS spectra and online MS/MS spectra, most of the standard antibody Fabs can be confidently assigned. Representative high-resolution MS spectra were demonstrated for 4 light chains and 4 heavy chains (Fig. 2C). Our analysis demonstrated the feasibility of separating reduced intact Fab fragments in complex samples such as serum autoantibodies.

Different dissociation methods available on commercial orbitrap instruments were applied to the online fragmentation of parent ions, including collisional induced dissociation (CID), higher-energy collisional dissociation (HCD), and electron transfer dissociation (ETD). The combination of different dissociation methods can significantly improve the sequence coverage of reduced Fab fragments. The representative Fab fragment was well characterized with 51% residue cleavages (67% for light chain and 40% for Fab heavy chain) (Fig. S2). All of the MS/MS spectra used for Fab identification were manually checked and confirmed.

**Figure 3:**
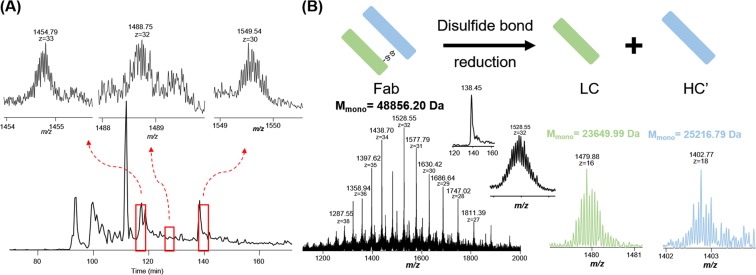
Light chain and heavy chain pair analysis using reduced and non-reduced Fab fragments. (**A**) The base peak chromatogram of non-reduced Fab mixture with selected MS spectra of three intact Fabs; (**B**) An example of a light chain and heavy chain pair. High resolution mass spectra of intact Fab and its corresponding light chain and heavy chain were shown above with detected accurate masses. M_mono_ represents the monoisotopic mass.

The analysis of Fabs with proper heavy and light chain pairing is crucial for antigen-binding studies of antibodies and this cannot be achieved with reduced samples. Therefore, the intact Fab fragment mixture (no reduction) was also analyzed using the UPLC-TD-HRMS platform. We have optimized our current Velos Orbitrap Pro’s performance, and were able to partially resolve intact non-reduced Fabs (*e.g.*, M.W. ~48 kDa). Specifically, we optimized the pressure in the FT instrument (1E-10 Torr or lower) and the related trapping time. We also optimized the AGC settings (optimized for different samples and dissociation methods) and accumulation time (1000 ms for full MS, 500 ms for MSn) to resolve these proteins. The results (Fig. 3A) indicated that intact non-reduced Fabs can be efficiently separated and analyzed using our UPLC-TD-HRMS platform. The detected masses from the non-reduced samples were used to pair the light chain and heavy chain masses detected from the reduced samples (Fig. 3B). Specifically, the detected light chain mass list and heavy chain mass list were manually compared to the detected intact Fab mass list. The light chain and heavy chain were considered as a pair if the sum of their masses minus 10 Da (coming from the reduction of 5 disulfide bonds) had a match in the intact Fab mass list with the possible mass shifts resulting from the mispicking of isotopic peaks (*e*.*g*., −1 Da, 1 Da). In the future, bioinformatic tools can be developed for more accurate and efficient pairing determination. The pairing results further need to be verified by sequencing information from the identified light chain, heavy chain, and intact Fab. Our results demonstrate that the developed UPLC-TD-HRMS platform is capable of separating and characterizing antibody Fab mixtures (both reduced and non-reduced) with high similarities, which can be applied to analyze enriched Fabs from human serum samples.

### UPLC-TD-HRMS analysis of monoclonal antibodies in human serum

**Figure 4:**
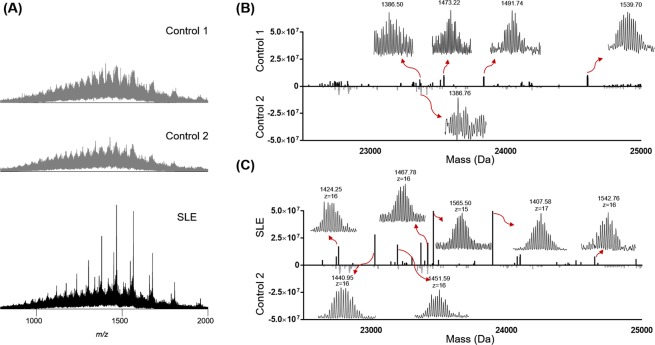
The UPLC-TD-HRMS analysis of human serum antibodies. (**A**) Summed mass spectra of two control samples (top) and the SLE serum sample (bottom). Our results suggested that serum antibody Fabs are difficult to resolve without separation. (**B**) is the comparison between two control samples using deconvoluted LC-MS mass features from 22,500 Da to 25,000 Da; (**C**) is the comparison between one control sample and one SLE serum sample using deconvoluted LC-MS mass features from 22,500 Da to 25,000 Da. Some of the high resolution mass spectra of the deconvoluted mass features are also shown above.

With the optimized platform, we analyzed several human serum samples and characterized higher abundance mass features that were putative autoantibodies in the serum with the ability to determine the light chains and heavy chains. Three serum samples were obtained from a SLE patient at different time points and the control samples were obtained from two healthy control individuals (autoantibody negative). All of the serum samples were purified using Protein A beads to enrich the antibodies from serum. After the enrichment, the samples were papain-digested and Fc portions were removed by Protein A beads. The purified Fab mixtures were reduced with TCEP and analyzed using the 1D UPLC-TD-HRMS platform. We first evaluated the summed MS spectra among the different samples (Fig. 4A). For control samples, the summed spectra showed a wide range of unresolved normal-distributed peaks, which makes the direct analysis unachievable^33^. For the SLE sample, several prominent mass features were observed, indicating the possible presence of autoantibodies. However, these features cannot be distinguished without further separations.

The UPLC-MS datasets were then deconvoluted to generate a “bird’s-eye” view of the total autoantibody composition in patient serum samples. Fig. 4B showed deconvoluted mass features between 22,500 Da to 25,000 Da, representing the molecular weight range of both Fab heavy chains and Fab light chains. In the control samples, most of the deconvoluted mass features have low intensities and are not well-resolved (*i*.*e*., less than 1E5 with the S/N less than 2). In control sample 1 (Fig. 4B), we observed 4 mass features with relatively good S/N ratios. We manually averaged the related scans for these mass features, and all of them have good isotopic distributions indicative of putative Fab chains. It is unlikely these putative Fab chains are from autoantibodies because these control serum samples tested negative against all known autoantigens, but they are likely from ongoing immune responses. Moreover, the measured intensities of these putative Fab chains are relatively low (*i*.*e*., less than 2E5). We further compared one of the SLE serum samples with one of the control samples (Fig. 4C). In the SLE serum sample, 122 mass features with S/N > 5 were identified among which 40 mass features were confidently detected with the total intensity larger than 1E6. All of these mass features were manually evaluated to ensure that they stood out from the serum antibody background.

**Figure 5:**
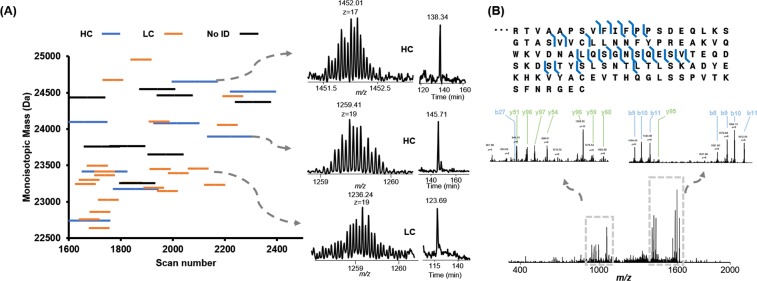
MS/MS identifications of putative Fab fragments in a SLE patient serum sample. (**A**) Plotting of the distribution of the deconvoluted mass features against the LC elution time. High resolution of the mass sepctra were displayed and their corresponding EICs were drawn by extracting the *m/z* of most abundant *m/z* ± 0.5. (**B**) MS/MS identification of one kappa 1 immunoglobulin light chain. The fragment ions (b and y ions) were labeled on the MS/MS spectra.

The Fab production process described above was performed on duplicate samples of SLE serum collected in year 3 and analyzed by the developed platform. All of the detected mass features were evaluated manually. From the result, the platform was proven to be highly reproducible between duplicates where run 1 and run 2 of this sample shared 39 detected mass features out of 47 and 43 detected mass features, respectively. Overall, we confidently detected 86 unique mass features with intensity larger than 1E6 in the putative Fab range in three samples from 3 different years from the same patient. We further plotted the deconvoluted mass features against the LC elution time (Fig. 5A**)**. Mass spectra and EICs of two representative detected mass features are also shown. Baseline resolved monoisotopic distribution of detected mass features were achieved and the EICs showed the high resolution of the UPLC-TD-HRMS platform even with extremely complex background serum antibodies.

In order to identify the detected mass features, the collected MS/MS raw data were processed with TopPIC and IMGT database^28^ was used to search the data. The LC run in Fig. 5 was used as the test dataset. In this dataset, a total of 20 PrSMs were confidently identified as either light chains or heavy chains of intact Fabs. After manually checking the MS/MS spectra, we noticed a common motif, VFIFPP (Fig. 5B), was confidently identified in all MS/MS spectra of the identified light chains with fully cleavage site coverage, which is a shared sequence segment in human immunoglobulin kappa light chain. Therefore, to facilitate the identification confidence, we apply it as the criteria of light chain mass features in our manual validations. Thus, in the filtered PrSMs, a mass feature was assigned as a light chain if more than two product ions of VFIFPP were observed. We manually analyzed the identified CID MS/MS spectra and confirmed many of them as intact Fab light chains in the SLE serum sample (examples in Supplementary Fig. S3). In total, 47 unique light chains and 16 unique heavy chains were identified in the three samples from one SLE patient collected over three successive years.

## Discussion

We developed and optimized a UPLC-TD-HRMS platform for the separation and characterization of the Fab fragments of autoantibodies in human serum. The UPLC-TD-HRMS platform provided sufficient separation of complicated antibody Fab mixtures and comprehensive characterization of Fab for light chain/heavy chain classification with high resolution, high run-to-run reproducibility, and in a high-throughput manner. Using our platform, we were able to identify a total of 47 light chains and 16 heavy chains from SLE patient serum. To our knowledge, this work is the first, to date, top-down MS demonstration of the human serum autoantibody pool analysis enabling the classification of light chains and heavy chains, which gives us a ‘bird’s-eye’ view of the complexity of human serum autoantibodies. Our results showed the potential of using high resolution separation methods coupled to high resolution and highly sensitive mass spectrometry detection to help the understanding of human serum autoantibodies. However, for in-depth profiling and understanding the key question of how many monoclonal antibodies are in SLE sera, we need to develop high resolution and high sensitive separation techniques that can efficiently separate highly homologous monoclonal antibodies from the background signal. Future studies can be done to improve the LC separation resolution using various orthogonal separation techniques such as hydrophilic interaction chromatography (HILIC), ion-exchange chromatography (IEC), and two-dimensional separation using high-pH and low-pH reversed phase liquid chromatography (2D pH RPLC) separations^34–36^. We note that currently selected patient samples are from a patient with advanced disease. More sensitive separation approaches such as capillary electrophoresis^37^ and targeted approaches can be incorporated for early stage autoantibody detections. High-end mass spectrometers (*i*.*e*., 21 T FTICR-MS) with different fragmentation techniques, such as front-end electron transfer dissociation^38^ and ultraviolet photodissociation (UVPD)^13^, can also be applied to increase the mass detection range and protein sequence coverage.

One of the challenges of serum autoantibody sequencing and characterization is the complexity of human antibody repertoire. Antibody gene sequencing of B-cells from particular patient can be performed to enlarge the existing database for more targeted identifications of antibodies in human serum samples. In addition, we also observed some of the MS/MS spectra with good quality fragmentation were not identified which might be due to the incomplete database. We have been successfully applying integrated top-down and bottom-up approach for comprehensive de novo sequencing of standard antibodies^28,39^. In future work, the optimization of this sequencing tool as well as other related software will be done to expand the identifications. Our results, overall, demonstrate the ability of our platform to perform top-down MS analysis on complicated human serum samples and to detect antibodies developing in patient serum over years, which gives it the possibility to monitor the development of antibodies during autoimmune disease progression.

## Materials and Methods

### Materials and Reagents

LC/MS CHROMASOLV® grade isopropanol (IPA), acetonitrile (ACN), LC-MS grade water, acetic acid (HAc), and phosphate buffered saline (PBS) were purchased from Sigma-Aldrich (St. Louis, MO). Pierce™ Trifluoroacetic Acid (TFA), Bond-Breaker™ TCEP solution, Protein A/agarose beads, and papain were obtained from ThermoFisher Scientific (Hanover Park, IL). The packing materials for packing C5 (Jupiter particles, 5 µm diameter, 300 Å pore size) was purchased from Phenomenex (Torrance, CA). Amicon concentrators (10 kDa and 30 kDa) were obtained from Millipore (Burlington, MA).

### Human subjects

SLE and healthy control plasma samples were obtained in accordance with the Helsinki Declaration and were approved by the Institutional Review Board at the Oklahoma Medical Research Foundation and patients agreed with the informed consent to participate. Blood was collected via venipuncture into ACD vacutainers (BD Biosciences, San Jose, CA), spun and the plasma was removed and stored at −20 °C until used. The plasma from this particular SLE patient (de-identified sample) contains ~0.5 mg/mL of anti-Sm, as well as smaller quantities of anti-nRNP, anti-Ro and anti-La (data not shown).

### Monoclonal antibodies

Fully human, full-length monoclonal antibodies were produced by the Human antibody core facility at the Oklahoma Medical Research Foundation as previously reported^40^. These antibodies were obtained from single cell-sorted antibody secreting cells or naïve B cells and are expressed with human IgG1 heavy chains and kappa or lambda light chains.

### Sample preparation

*Escherichia coli* cell lysate proteins were obtained from the BL21 strain grown in house. Cell lysate was obtained by bead-beating with zirconia silica beads^34^. Aliquots of protein solutions were stored at −80 °C until further use. Protein A/agarose beads were used for the antibody purification from serum samples. In detail, protein A beads were incubated with diluted plasma (3–5 ml total plasma, diluted 1:5 in 1 × PBS) at 4 °C overnight. The antibodies were eluted with 0.1 M glycine-HCl (pH 2.7) and concentrated using Amicon concentrators (30 kDa cutoff). The purified IgG fractions, as well as standard monoclonal antibodies were then digested using insoluble papain suspension with incubation at 37 °C for 4 hours. After the incubation, protein A/agarose beads were used for the removal of Fc fragments from the antibody digests with the same conditions as previous described. Fab fragments from either plasma or monoclonal antibodies were then concentrated to >1 mg/mL total protein concentration with Amicon protein concentrators (10 kDa cutoff). The Fab fragments were reduced by reacting with 1 μL of 0.5 M TCEP for 10 minutes at room temperature prior to the UPLC-TD-HRMS analysis.

### UPLC-TD-HRMS

An in-house packed nano-flow capillary RPLC-C5 column (5 μm, 75 μm × 100 cm) was used on a custom modified UPLC (maximum pressure 14,000 psi) system. The mobile phase A was 0.01% TFA, 0.585% HAc, 2.5% IPA and 5% ACN in water, and the mobile phase B was 0.01% TFA, 0.585% HAc, 45% IPA and 45% ACN in water. Ten micrograms of *E*. *coli* lysate proteins, 8 micrograms of 12-antibody-Fab mixture, or 4 micrograms of purified human serum antibody Fab samples were loaded on the column for the top-down MS analysis individually. A gradient from 10% to 70% of mobile phase B over 70 minutes or 280 minutes at a flow rate of 400 nL/min was applied for the separation and the column was regenerated by flushing with 90% of mobile phase B for 10 minutes and equilibrated to 100% of mobile phase A. The nano-LC column was directly coupled to an LTQ Orbitrap Velos Pro mass spectrometer (ThermoFisher Scientific, Bremen, Germany) for online MS/MS analysis with a custom designed nano-ESI interface under positive mode. The electrospray voltage was set to 2.6 kV and the heated inlet capillary temperature was optimized to 250 °C. MS data were collected at the resolving power setting of 100,000 (at *m/z* 400) with two micro scans. MS/MS acquisition was performed by selecting the top five most abundant precursor ions in the full MS scan using collision induced dissociation (CID), higher-energy collisional dissociation (HCD), or electron-transfer dissociation (ETD). The CID was performed with the normalized energy of 35% with an isolation window of 3.0 *m/z*. HCD was performed with 22% normalized energy, and the ETD was performed with the activation time set to 20 ms. The isolation window was set to 4.0 *m/z* for HCD and ETD. The MS/MS data were obtained at a resolving power setting of 60,000 (at *m/z* 400) with two or three micro scan counts. Ions with less than 4 charges were rejected for the selection of MS/MS scans. The maximum injection time for a full mass scan and a MS/MS scan were set to 1000 ms and 500 ms, respectively. The AGC target was set at 6 × 10^5^ for full mass scans, and 3 × 10^5^ for MS/MS scans for CID runs, and 1 × 10^6^ (full mass scans), 1 × 10^6^ (MS/MS scans) for HCD and ETD runs. All of the data were collected with the Xcalibur 3.0 software (Thermo Fisher Scientific, Bremen, Germany).

### Data analysis

The MS raw data were converted to centroid mzXML files with msconvert (a tool in ProteoWizard^41^) and deconvoluted with MS-Deconv^42^. A constant region sequence database (55 heavy, 5 kappa, and 13 lambda sequences) was downloaded from the IMGT database^28^. The deconvoluted data were searched against the sequence database separately using TopPIC^43,44^, in which the error tolerances for precursor and fragment masses were 15 ppm and at most 2 unknown mass shifts were allowed in a proteoform spectrum-match. Other parameter settings in TopPIC can be found in the supplemental information (Table S1). All of the MS/MS spectra were manually evaluated to match the signature sequence tag for LC/HC classification. All the identifications are manually evaluated using ProSight Lite^45^.

## Supplementary information

supplementary Figures
