# Supplementary material for: Top-down Mass Spectrometry Analysis of Human Serum Autoantibody Antigen-Binding Fragments
Source: Sci Rep. 2019 Feb 20;9:2345. doi: 10.1038/s41598-018-38380-y (PMC6382847; doi:10.1038/s41598-018-38380-y)
Supplement: Supplementary file 1 — supplementary Figures [file 41598_2018_38380_MOESM1_ESM.pdf]

Top-down Mass Spectrometry Analysis of Human Serum Autoantibody Antigen-Binding Fragments

Zhe Wang[1], Xiaowen Liu[2], Jennifer Muther[3], Judith A. James[3,4], Kenneth Smith[3,*], and Si Wu[1, *]

1. Department of Chemistry and Biochemistry, University of Oklahoma, Norman, OK 73019

2. School of Informatics and Computing, Indiana University-Purdue University Indianapolis, Indianapolis, IN 46202

3. Department of Arthritis and Clinical Immunology, Oklahoma Medical Research Foundation, Oklahoma City, OK 73104

4. Departments of Medicine and Pathology, University of Oklahoma Health Sciences Center, Oklahoma City, OK 73104

*To whom correspondence should be addressed.

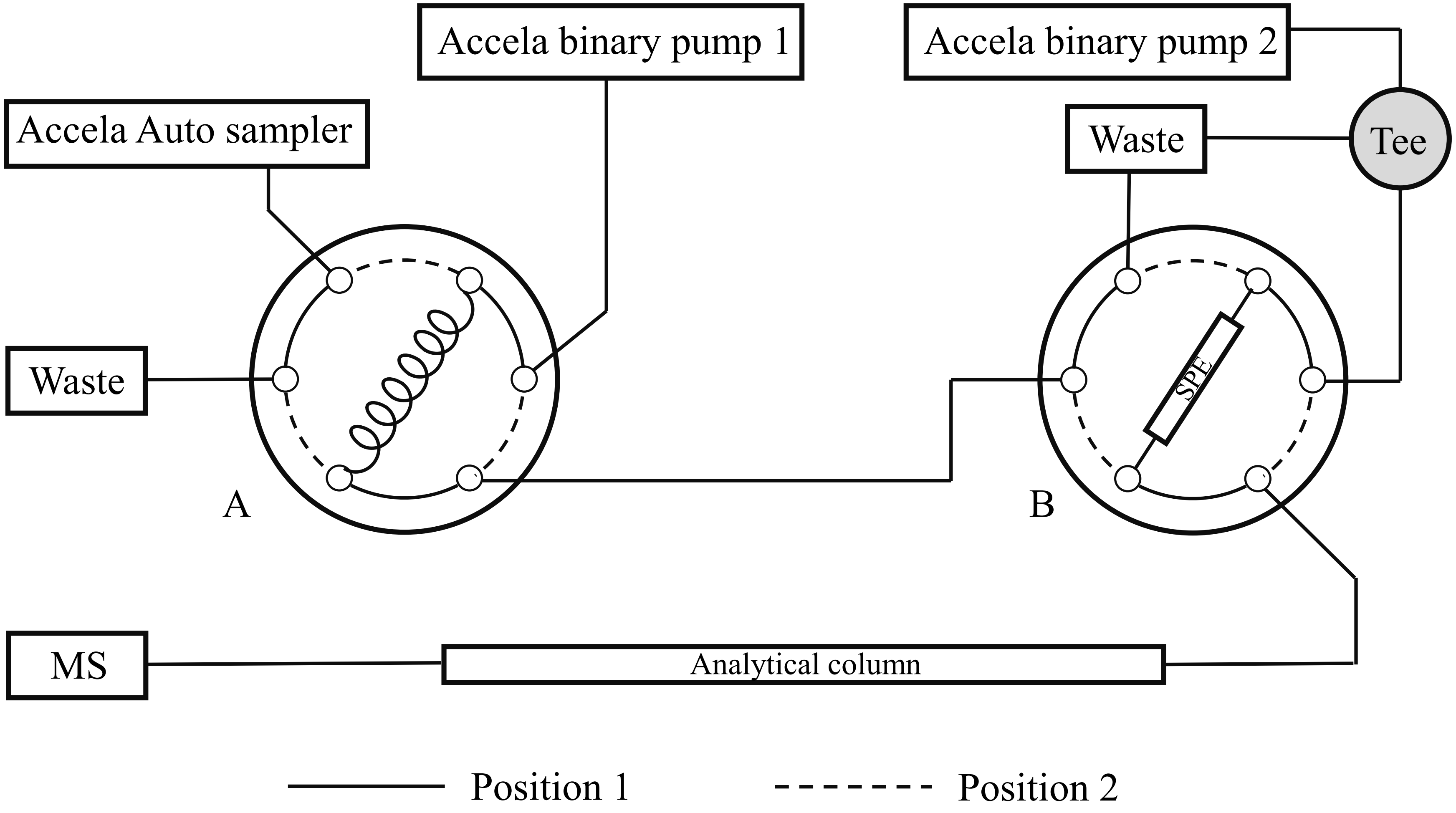

Figure S1. The setup of the modified LC system. Two Accela binary pumps are used for the sample trapping and LC separation. The sample loop volume is 25 µL. The SPE is a C4 trapping column made of the same material as the separation column, with 150 µm I.D., 10 cm length.

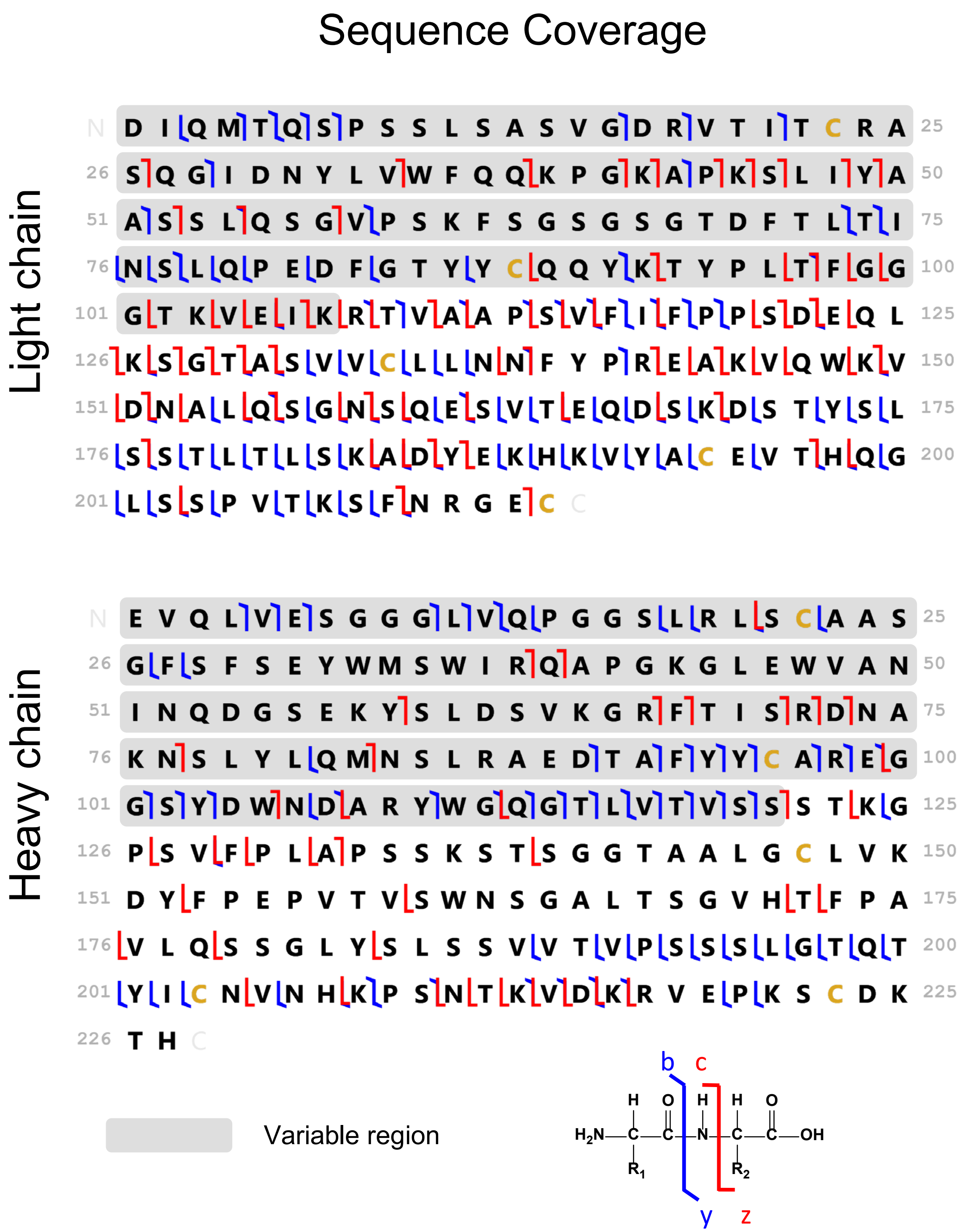

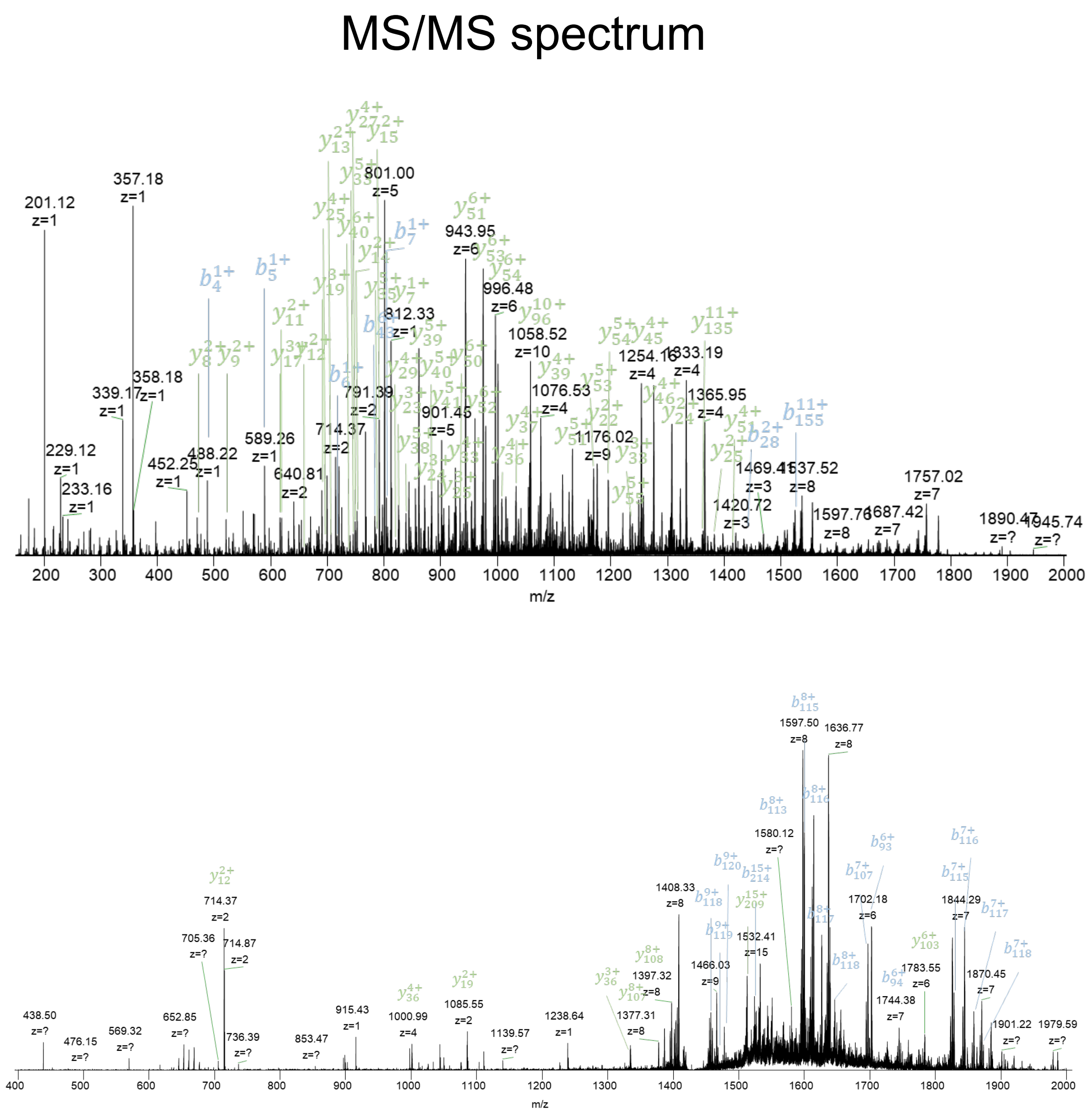

Figure S2. The identifications of the Fab light chain and Fab heavy chain of an antibody. Different fragmentation methods (e.g. HCD and ETD) were used to improve the sequence coverage. Examples of the MS/MS spectra of Fab light chain and Fab heavy chain were shown in the figure.

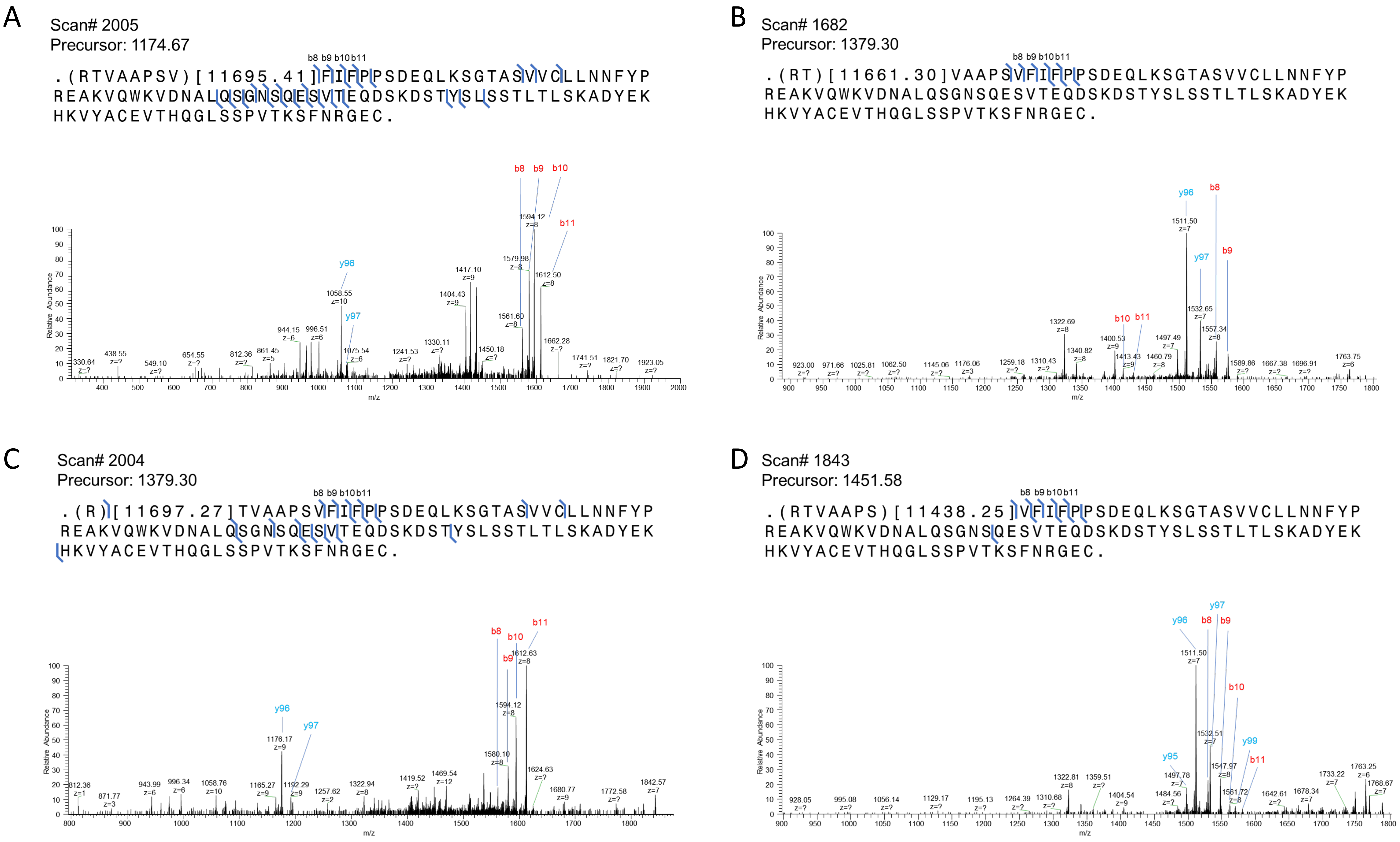

Figure S3. Examples of identified light chains with annotated MS/MS spectra

Table S1: Parameter settings of TopPIC in the analysis of the top-down MS/MS data of human serum samples

**Parameter**	**Value**
Fragmentation method	FILE
Fixed modifications	None
N-terminal forms of proteins	NONE, NME, NME+ACETYLATION
Using a decoy database	No
Error tolerance	15 ppm
Maximum number of unexpected mass shifts in a proteoform spectrum-match	2
Spectrum level cutoff type	E-value
Spectrum level cutoff value	0.01
Number of combined spectra	1
E-value computation method	Lookup table
